# Mesenchymal stem cells and their extracellular vesicles: new therapies for cartilage repair

**DOI:** 10.3389/fbioe.2025.1591400

**Published:** 2025-04-24

**Authors:** Hongwei Yang, Haochen Yang, Qin Wang, Hanzhen Ji, Tianmei Qian, Yusen Qiao, Junfeng Shi, Meng Cong

**Affiliations:** ^1^ Department of Orthopedics, Affiliated Nantong Hospital 3 of Nantong University, Nantong, China; ^2^ Key Laboratory of Neuroregeneration of Jiangsu and Ministry of Education and Co-Innovation Center of Neuroregeneration, Nantong University, Nantong, China; ^3^ School of Medicine, Nantong University, Nantong, China; ^4^ Engineering Research Center of Integration and Application of Digital Learning Technology, Ministry of Education, Beijing, China; ^5^ Department of Orthopaedics, The First Affiliated Hospital of Soochow University, Suzhou, China

**Keywords:** mesenchymal stem cells, extracellular vesicles, cartilage injury, cartilage regeneration, regenerative therapy

## Abstract

Cartilage is crucial for joints, and its damage can lead to pain and functional impairment, causing financial burden to patients. Due to its weak self-repair, cartilage injury control is a research focus. Cartilage injury naturally with age, but mechanical trauma, lifestyle factors and certain genetic abnormalities can increase the likelihood of symptomatic disease progression. Current treatments for cartilage injury include pharmacological and surgical interventions, but these lack the ability to stop the progression of disease and restore the regeneration of the cartilage. Biological therapies have been evaluated but show varying degrees of efficacy in cartilage regeneration long-term. The mesenchymal stem cell (MSC) therapy attracts attention as it is easily harvested and expanded. Once thought to repair via differentiation, MSCs are now known to secrete extracellular vesicles (EVs) paracrinely. These EVs, rich in bioactive molecules, enable cell communication, boost growth factor secretion, regulate the synthesis and degradation of extracellular matrix (ECM), and modulate inflammation, vital for cartilage repair. However, further research and clinical validation are still required for the application of MSC and MSC-EVs. This review highlights the current state of research on the use of MSC and MSC-EVs in the treatment of cartilage injury. It is hoped that the review in this paper will provide valuable references and inspiration for future researchers in therapeutic studies of cartilage repair.

## 1 Introduction

The cartilage consists of dense extracellular matrix (ECM) and chondrocytes embedded in it, which have functions such as lubrication, shock absorption and decompression. The cartilage contains no blood vessels, lymphatic vessels, and nerves, so the regeneration rate of chondrocytes is relatively slow, and its natural repair ability is very limited, making it susceptible to injury and degenerative disease, further leading to pain, dyskinesia and loss of function (Koh et al., 2020). In recent years, the incidence of articular cartilage injury has been increasing with the aging of the population (Muthu et al., 2023). Other risk factors for cartilage injury include mechanical trauma, genetic predisposition, lifestyle factors and certain metabolic disorders (Xu et al., 2022).

The treatment of cartilage injury often becomes a more complex issue, and the type and degree of injury determine the differences in treatment options, such as non-steroidal anti-inflammatory drugs and chondroitin sulphate for oral administration, sodium hyaluronate and joint replacement surgery performed in patients with severe cartilage injury or advanced osteoarthritis (OA) (Zhou et al., 2020). Current treatments of cartilage injury aim to manage symptoms and minimize disability, but both pharmacological and surgical interventions can lead to complications, can be costly and have questionable efficacy. Neither treatment option is capable of targeting the underlying cause of injury, and both lack the ability to hinder the progression of disease or regeneration to restore the precedent functionality of the cartilage. Various biological therapies, including the use of growth factors and platelet-rich plasma (PRP), have been evaluated in both preclinical and clinical studies, but many results have been underwhelming (Gazendam et al., 2021; Belk et al., 2021). Preliminary preclinical studies have attempted to use gene editing (Chaudhry et al., 2022; Xu et al., 2023), or to improve current surgical methods using biomaterials, but studies of this kind are still in their infancy (Chen et al., 2023).

Mesenchymal stem cell (MSC) based therapies, both allogeneic and autologous, are an attractive method due to their ability to target many of the pathways that result in cartilage injury (Le et al., 2020). There has been progress with these studies, but there are concerns about a low survival rate of transplanted cells. Studies on the mechanism of stem cell-based therapies have provided increasing evidence that extracellular vesicles (EVs) secreted by MSCs are responsible for the regenerative properties and efficacy in treating cartilage injury (Yang et al., 2024). While initially considered a waste product, EVs have recently been highlighted for their role in intercellular communication (Ding et al., 2023; Tieu et al., 2020). EVs show pronounced therapeutic competence for tissue regeneration through the maintenance of their endogenous stem cells, their ability to modulate the immune response and inhibit apoptosis, stimulation of angiogenesis and their enhancement of regenerative phenotypic traits (Tieu et al., 2020). The mesenchymal stem cell (MSC) derived EVs (MSC-EVs), specifically, present a significant opportunity for the safe and effective treatment of cartilage injury because of their ability to maintain the therapeutic benefit of their origin cells without the risks associated with MSC-based therapies (Abreu et al., 2022; Nguyen et al., 2021; Lu V. C. et al., 2021). This review highlights the current studies being conducted on MSC and MSC-EVs in the treatment of cartilage injury.

## 2 Cartilage injury

### 2.1 Subsection structure of the cartilage

**FIGURE 1:**
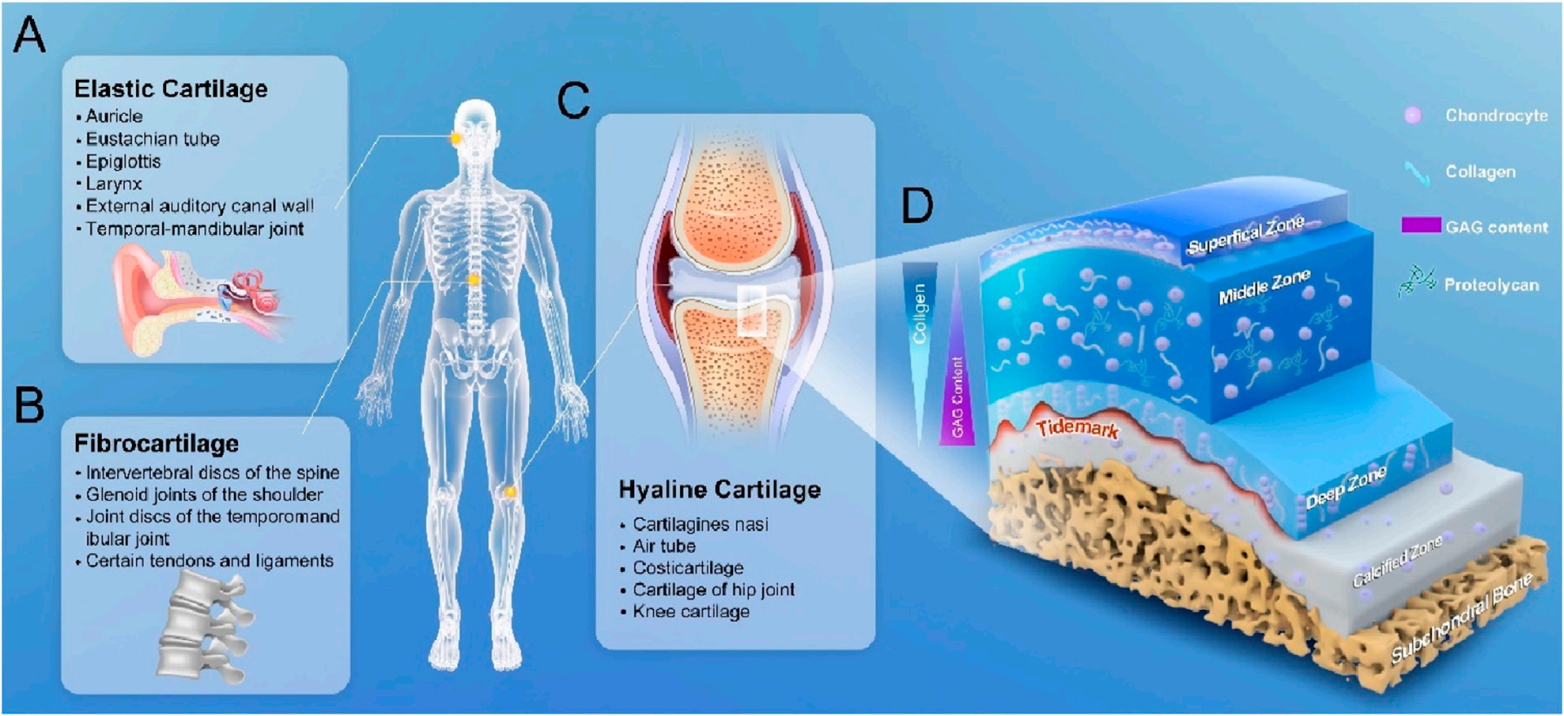
Classification of cartilage and 3D anatomical diagram of articular cartilage. **(A)** elastic cartilage, which is distributed in the areas such as auricle, walls of the external auditory canal and eustachian tube, epiglottis and larynx. **(B)** Fibrocartilage, which is distributed in the areas such as spinal intervertebral discs, glenoid cavity in shoulder, and temporal-mandibular joint. **(C)** Articular cartilage, which is also known as hyaline cartilage and distributed in the surface areas of the trachea bronchi, bones and joints. **(D)** 3D structure of a normal articular cartilage: four zones of the cartilage are highlighted: the superficial zone where the flattened chondrocytes are located, the middle zone where the elongated chondrocytes are located, the deep zone where the chondrocytes are arranged in columns at the bottom, and the calcified zone.

Cartilage, as a tough fibrous connective tissue plays an important supportive and protective role in the musculoskeletal system. Cartilage can be divided into three types: hyaline cartilage, elastic cartilage and fibrocartilage, of which, the hyaline cartilage is the most common type of cartilage in the human body. In its fresh state, the hyaline cartilage has a semi-transparent appearance and constitutes the normal articular cartilage (Figures 1A–C) (Krishnan and Grodzinsky, 2018). With a lubricated surface, the articular cartilage is located on the surface of a movable joint, and serves as a shock absorber, thus reducing friction between adjacent bones, and transferring a mechanical load to the deep subchondral bone plate, which facilitates bone movement (Lin and Klein, 2021). The articular cartilage contains no blood vessels, lymphatic vessels, or nerves, and consists of chondrocytes embedded in a dense extracellular matrix (ECM), and the chondrocytes are the predominant cell type in cartilage. Chondrocytes account for about 1%–10% of the total cartilage volume and are responsible for the synthesis and degradation of all ECM and maintenance of a balance between synthesis and degradation. ECM primarily consist of water (accounting for 68%–85% of the total wet weight), collagen (60%–86% of the dry weight), proteoglycans (primarily aggrecan, 15%–40% of the dry weight), and other lesser non-collagenous proteins (including link protein, fibronectin, cartilage oligomeric matrix protein) and the smaller proteoglycans [biglycan, decorin and fibromodulin (Eschweiler et al., 2021)]. The fluid in the joint cartilage can not only transport nutrients to the cartilage cells, but also provide lubrication to the joint surface. The most abundant collagen in articular cartilage is type 2 collagen [Type II Collagen (Col II), accounting for 90%–95% of the entire collagen content], which forms microfibrils, protofibrils, and mature collagen fibers interwoven with proteoglycan aggregates. Proteoglycan is a special class of glycoproteins that are formed by covalently linking one or more glycosaminoglycans (GAGs) to a core protein. In addition to GAGs, proteoglycans also have some N- and/or O-linked oligosaccharide chains. Proteoglycan are distributed not only in the ECM, but also on the cell surface and in intracellular secretory granules, and form larger proteoglycan aggregates with different compositions and functions of GAGs through connecting and interacting with proteins and hyaluronic acid chains. The main types of GAGs in articular cartilage are hyaluronic acid, chondroitin sulphate, keratan sulphate and dermatan sulphate. The anionic GAGs can attract cations from water, providing the articular cartilage with osmotic properties (Mead et al., 2022). The collagen fibre network and the attached proteoglycan aggregates together contribute to the resistance of cartilage to compression.

From a developmental perspective, the articular cartilage is formed by the differentiation of corresponding MSCs, which are guided by relevant signals to aggregate and start synthesizing the matrix. As time progresses, once a large number of these cells have been aggregated and dispersed in the surrounding matrix, they become more organized, and are thus defined as cartilage. At this time, the morphologies of the cells have also changed greatly, and tend to show a more characteristic spherical shape. As time progresses, along with the gradual maturation of the cartilage, the number of cells with multi-differentiation potential is gradually reduced, and new chondrocytes will lose their abilities to migrate, proliferate, and participate in the repair of cartilage injury, and these basic physiological characteristics severely limit the repair potential of articular cartilage. Furthermore, mature chondrocytes themselves have only a limited ability to increase the synthesis of their surrounding matrix, and there is a programmed cell degeneration throughout the microenvironment, which can limit the abilities of chondrocytes to respond to stimuli and synthesize certain types of proteoglycans (Decker, 2016). Although the basic components of the whole articular cartilage are the same, there are obvious regional differences in the structure, component concentration and morphology of chondrocytes in the vertical direction, and the overall structure of articular cartilage is divided into four demarcated zones. These zones are classified as 1) superficial/tangential zone, 2) intermediate/transitional zone, 3) deep/radial zone, and 4) calcified zone (Figure 1D). These four zones account for 10%–20%, 40%–60%, 30%–40%, and 10%–20% of the thickness of articular cartilage, respectively (Wei and Dai, 2021). From the surface to the deeper layers of articular cartilage, the content of proteoglycan aggregates tends to increase, the contents of water and chondrocytes gradually decrease, and the concentration of collagenous protofibers remains almost constant. In the superficial zone, Col II fibers are relatively thin, closely arranged and parallel to the articular surface, and chondrocytes are flatly distributed in the interstices of the collagen fibers, and this zone is essential for maintaining the lubricating and tensile properties of the tissue in contact with the synovial fluid (Carballo et al., 2017). The researchers have focused their studies on the upper layer where the density of articular chondrocytes is lowest, as this layer has been shown to possess stem cell-like properties. The nomenclature of this region is evolving and is often referred to as the cartilage layer, and the superficial zone (SZ). It is so named because it is a relatively distinct translucent layer on the articular surface. This transparent thin membrane can be mechanically removed from the underlying cartilage. Removing this layer will increase the permeability of the tissue and may increase a load on the macromolecular framework during the compression resistance. Disruption or remodeling of dense collagen matrix in the SZ is the earliest detectable structural change in experimentally induced degeneration of articular cartilage, where a dense network of collagen fibers forms a “skin” that may restrict the influx of macromolecules (antibodies, proteins) and the outflow of large cartilage molecules. This layer also acts as a macromolecular filter membrane to form a barrier between synovial fluid and cartilage, thus SZ can effectively isolate cartilage from the immune system. This layer may play a very important role in lubricating the joint and protecting the underlying large cartilage from loading effect. The cells in the SZ secrete a lubricating proteoglycan, lubricin, which also plays a role in lubricating the joint and effectively protecting articular cartilage from wear and tear (Sun et al., 2022). The chondrocytes in the SZ diminish with age, and their injuries and erosion are the earliest observed histological changes in degenerative cartilage disorders. The largest intermediate zone is located immediately below the superficial layer, where the collagen fibers are thicker and randomly arranged and the cells are rounder. The intermediate zone serves as an anatomical and functional transition between the superficial and deep zones, and is the first line of defence against the compressive forces from the articular surface. In the deep zone, the collagen fibers are bundled together as rigid fibers perpendicular to the cartilage surface, and the chondrocytes are parallel to the collagen fibers in a columnar direction, and can appear as several cell clusters. This zone contributes mostly to the resistance to compression during body movement. The calcified zone is located at the bottom, a basophilic line that demarcates the boundary between non-calcified and calcified cartilage. Calcified cartilage and subchondral bone, with an expression of type X collagen, effectively separate the articular cartilage from the bone.

### 2.2 Types and clinical diagnosis of cartilage injury

Articular cartilage consists of chondrocytes, collagen, proteoglycans and water, and has functions such as lubrication, shock absorption and decompression. Unlike other tissues, the cartilage has a very limited blood supply of its own and a slow rate of cellular regeneration, so its natural repair capacity is more limited, making it susceptible to injury and degenerative disease, thus further leading to pain, dyskinesia and loss of function (Hu et al., 2021). According to the injury depth, the articular cartilage injuries can be classified into the following three types: 1) partial-thickness cartilage defects (PTCDs): PTCDs on the articular surfaces have a injury depth not exceeding the calcified layer of the cartilage; 2) full-thickness cartilage defects (FTCDs): FTCDs have a injury depth exceeding the calcified layer of cartilage (Sun et al., 2022; Nammour et al., 2024). PTCDs are the most common complication of cartilage degeneration. More than 60% of the knee joints examined by arthroscopy have joint defects, most of which are chronic PTCDs that are difficult to cure; once the cartilage injury occurs, if it is not treated promptly and appropriately, the injury may continue to increase, leading to the destruction of the synthesis and degradation metabolism in the cartilage, eventually leading to FTCDs and subsequent OA (Tsuruoka et al., 2011). Both PTCDs and FTCDs can cause new cartilage injury in knee OA; compared to FTCDs, the subchondral bone is not injured, and there is no blood supply and bone marrow exudate in PTCDs; when PTCDs occur, the microenvironment around the injury is not suitable for bone marrow stem cells to adhere, thus the self-repairing capability is completely lost, and the symptoms are often more severe than those of FTCDs. Therefore, repairing PTCDs is crucial for preserving deeper or surrounding healthy cartilage, and can also reduce or prevent the onset of OA. Articular cartilage injuries may occur as a result of repetitive impact, trauma, or progressive mechanical degradation caused by a variety of activities or events, which manifest themselves in specific ways. The isolated traumatic cartilage injury or osteochondral defect occurs more frequently in younger patients, whereas the degenerative cartilage lesion is rarely seen in younger patients. Currently, clinicians often fail to notice significant changes in early articular cartilage injury in patients with chronic degeneration, only detecting the injury after clinical symptoms appear. Asymptomatic cartilage damage is frequently discovered incidentally during arthroscopy. Over time, these cartilage injuries may worsen with age and contribute significantly to the development of osteoarthritis (OA). In addition, the articular cartilage surfaces subjected to impacts delivered by high-energy loads may undergo immediate changes in their cartilage surfaces, resulting in matrix loss, chondrocyte death, and subchondral bone injury. If the traumatic load is sufficiently high, it may lead to subchondral fractures and trabecular fragmentation. Some scholars have tried to create an animal model of articular cartilage injury on the articular surface, and their data show that the cell and matrix components are destroyed, and a large number of chondrocytes undergo apoptosis (Masson and Krawetz, 2020). The subchondral fractures and trabecular fragmentation can stimulate bone and cartilage repair. However, the injured articular injury is unbroken, there are currently no preventative therapeutic measures to alter the final outcome other than repairing the cartilage injury by changing the load at the joint site during the potential healing phase, and the repaired tissues rarely restore the mechanical and biological properties of natural articular cartilage. As time goes on, the clinical doctors may notice displacement or local degeneration of injured articular injury. Therefore, the injured articular cartilage should be monitored early, and establishing a recovery threshold for articular cartilage injury is an area that needs further research.

Molecular markers can be used to accurately record changes in the early stages of the disease and subsequent series of progression, and can exhibit a more intuitive response to surgeons (Bodaghi et al., 2023). The difficulty in diagnosing cartilage injury without specific markers is that the majority of the molecules involved in cartilage degradation and synthesis can still be detected in many tissues outside the target (affected) articular cartilage, making it difficult for current diagnostic methods to distinguish between affected and unaffected organs or tissues. In addition, the contents of articular cartilage molecules in the blood and urine do not always correlate with a localized change of articular injury. Before the development of appropriate markers, the common method for diagnosing cartilage injury in the clinic was based on symptoms, physical examination findings, and X-ray results, with MRI also serving as an auxiliary diagnostic tool. At the same time, cartilage injury rarely occurs in isolation and is often accompanied by ligament or meniscus injuries. X-rays can determine whether the joint space is narrowed. If the joint space narrowing is confirmed by X-ray examination, it is indicated that the patients have cartilage hyperplasia or injury. Artilage hyperplasia, as an abnormal increase in the number of chondrocytes in the cartilage tissue, may be a response by the body to repair the damaged cartilage. However, in some cases, the proliferated cartilage may not have the same function as normal cartilage, and it may exacerbate the degenerative process of the joint, affecting the normal movement and structure of the joint. This method has a lower diagnostic level for early cartilage injury. The milder cartilage injuries may only be displayed and documented as haemorrhage and oedema on magnetic resonance imaging (MRI), which are usually interpreted as signal changes in the subchondral bone, representing the injury site (Strickland et al., 2024). With the advancement of science and technology, the genetic screening with microarrays of selected activated genes is considered a more promising emerging technology. In view of this, it is necessary for us to further study the mechanisms of cartilage injury and identify corresponding key regulatory factors, and design a novel diagnostic equipment, so as to further increase the early diagnosis rate of cartilage injury, prevent and control the occurrence of OA, and improve the quality of life for patients.

### 2.3 Pathogenesis of cartilage injury

The joint contains cartilage, bone, synovium, ligaments, and fat pads, etc. The transfer and exchange of information and material between tissues and cells occur all the time. It is important to understand the transfer of these intercellular factors, develop appropriate treatment strategies, and ultimately prevent the occurrence of OA after cartilage injury.

Chondrocytes respond to injury or load within hours after it occurs. The injuries in the early stage are characterized by the activation of signaling pathways involved in osteogenesis and joint formation. It has been shown that the injured cartilage releases fibroblast growth factor 2 (FGF-2), a cytokine that promotes chondrocyte proliferation, induces phosphorylation of extracellular signal- regulated kinase (ERK), thereby protecting cartilage (Farooq et al., 2021). In addition, the transforming growth factor-β (TGF-β) and bone morphogenic protein (BMP) signaling pathways are rapidly activated when the skeletal injury and OA occur. TGF-β transduces its signals through the intracellular mediators *drosophila* mothers against decapentaplegic protein 2 (SMAD-2) and/or SMAD-3, which can promote the maintenance of relative stability of chondrocytes and induce the production of proteoglycans and Col II. On the other hand, BMP transduces its signals through SMAD-1, SMAD-5, and SMAD-8, which can stimulate the expressions of Col X, matrix metalloproteinase (MMP-13), and vascular endothelial growth factor (VEGF) in chondrocytes and promote the hypertrophic differentiation of chondrocytes (Wu et al., 2024). It is well known that Wnt signaling molecules regulate the proliferation, differentiation and growth of various cells. The Wnt/β-catenin signaling pathway is closely associated with inflammation and subchondral bone remodeling after cartilage injury (Sherwood et al., 2014). In mammals, the cartilage injury directly leads to a decrease in autophagy depending on the mammalian target of rapamycin (mTOR) and an increase in cell death (Lotz and Caramés, 2011). It has also been found that activation of phosphatidylinositol 3-kinase and protein kinase B (PI3K/Akt) and mitogen-activated protein kinase (MAPK) signaling pathways can inhibit NF-κB signal transduction pathway (NF-kB), and ultimately prevent chondrocyte apoptosis by inhibiting the production of reactive oxygen species (ROS) (Hossain et al., 2021). In addition to the above factors, the immune cells also play an important role in cartilage injury and repair. The inflammatory environment generated by cartilage injury is critical for chondrocyte death and hypertrophy, extracellular matrix decomposition, ectopic bone formation, and progression of cartilage injury to OA. The immune cells involved in cartilage injury and repair mainly include macrophages, osteoclasts, T cells, B cells, natural killer cells (NK cells) and dendritic cells (D cells) (De Lange-Brokaar et al., 2012). At present, it is unclear which cytokines are primary or secondary driving factors in the joint injury. However, the immune response is increasingly recognized as a key factor affecting the cartilage repair, which has both positive and negative regulatory effects on regeneration and repair process. The first immune cells to be recruited during injury are neutrophils, which secrete pro-inflammatory mediators and elastases and have the ability to re-phagocytose macrophages, D cells and NK cells, as well as inducing chondrocyte apoptosis and ECM degradation. Interferon-γis released upon activation of NK cells and helper T cells 1 (Th1), which can polarize the infiltrating macrophages to M1 macrophages. Then, M1 macrophages can secrete proinflammatory factors that interact with chondrogenic differentiation of MSCs to promote tissue fibrosis, MSCs will undergo an aberrant differentiation process, the chondrocytes start to be transformed or de-differentiated into fibroblast-like cells, which form the fibrocartilage with poor mechanical properties, thus leading to the degeneration of articular cartilage. Meanwhile the mastocytes can promote the degradation of ECMs to remove necrotic articular cartilage. During the repair process, the macrophages are polarized into M2 macrophages by IL-4 secreted by Th2 cells. M2 macrophages can secrete anti-inflammatory cytokines and chondrogenic cytokines, which can inhibit inflammation and promote articular cartilage repair. Therefore, it is required to perform multidimensional spatial and temporal regulation of the joint inflammatory microenvironment to facilitate articular cartilage regeneration (Katz et al., 2021; Li M. et al., 2021).

## 3 Current treatment of cartilage injury

### 3.1 Traditional non-surgical treatment of cartilage injury

Up to now, there are various clinical methods for repairing articular cartilage injury, which are mainly divided into two categories: non-surgical treatment and surgical treatment. Non-surgical treatments mainly include: 1) exercise, education, and weight loss, etc.; 2) oral non-steroidal anti-inflammatory drugs, glucosamine hydrochloride, and chondroitin sulphate, etc.; 3) intra-articular injections: glucocorticosteroids, sodium glutamate, and sodium hyaluronate, etc.; 4) physical therapy: radiofrequency energy (RFE), light therapy (LT), low-intensity pulsed ultrasound (LIPUS), and pulsed electromagnetic field (PEMF) etc (Solanki et al., 2021; Muhammad et al., 2018). All these methods can relieve pain and delay joint degeneration, but they cannot fundamentally repair the injured articular cartilage.

### 3.2 Surgical treatment of cartilage injury

Arthroscopic surgery is a minimally invasive procedure commonly used to treat early to mid-stage cartilage injuries. Through small incisions, surgeons insert an arthroscope into the joint to directly observe cartilage damage and perform cleaning, repair, or removal of damaged tissue (Brumat et al., 2022). High tibial osteotomy (HTO) and distal femoral osteotomy (DFO) are surgical procedures aimed at correcting lower limb alignment abnormalities (Sherman et al., 2018). By redistributing joint loads, these surgeries reduce pressure on damaged cartilage, promoting its repair and regeneration, thus preventing further cartilage injury. However, in the case of severe cartilage injuries—such as extensive cartilage detachment or significant joint deformity—arthroscopic surgery may not fully address the issue. Joint replacement surgery is typically considered when cartilage damage has progressed to an advanced stage, leading to severe loss of joint function and unrelievable pain. The postoperative recovery process is lengthy and involves certain risks, with satisfactory outcomes not always guaranteed (Katz et al., 2021). Some studies have considered that early treatment of cartilage injury is an important way to delay the onset of OA. The U.S. Food and Drug Administration (FDA) concluded that the microfracture procedure is an effective treatment method for smaller cartilage defects (<2 cm^2^). Bone marrow and plasma can pass through perforated channels to cover the injured articular cartilage, and BM-MSCs, cytokines, and platelets can be discharged from the bone marrow, which stimulate the regeneration of cartilage and repair inured articular cartilage. The microfracture procedure is favoured by a wide range of orthopaedic surgeons due to its simple one-stage procedure, limited invasiveness and good treatment effect (Hurst et al., 2009; Mithoefer et al., 2009). Another study has shown that after the application of microfracture procedure in treatment of patients with full-thickness cartilage defect, early histological repair is initiated by endochondral ossification at the deep puncture site (Hayashi et al., 2018). In addition, the endochondral osteogenesis can activate osteoclasts and induce cartilage reconstruction, and the cartilage regeneration occurs earlier than the subchondral bone regeneration. Meanwhile, it has also been found that the microfracture procedure can delay cartilage degeneration, regardless of the size of the lesion (Choi et al., 2023). However, some studies have shown that the microenvironment after microfracture procedure is not suitable for the differentiation of BM-MSCs. Ultimately, instead of a hyaline cartilage tissue, a relatively unstable fibrocartilage tissue is formed at the site of injury, it is unable to restore the normal cartilage morphology (Makris et al., 2015). The limitations of microfracture treatment in lesion size and long-term tissue function also necessitate the search for alternative methods of cartilage injury repair. The cartilage defects (2–4 cm^2^) can be treated with autologous osteochondral transplantation, autologous chondrocyte implantation, and allogeneic osteochondral transplantation, with no significant difference in the effectiveness of treatment among these three methods. For larger cartilage defects (>4 cm^2^), the autologous chondrocyte implantation or allogeneic osteochondral transplantation showed the best therapeutic effect, and some literatures have reported that the allogeneic osteochondral transplantation is a better option for the treatments of large osteochondritis dissecans lesions and post-traumatic osteochondral defects, thus reducing the risk of postoperative infection in the donor site and the problem of insufficient cartilage in the donor site (Krych et al., 2020).

In patients with trauma and cartilage defect, the surgeon’s goals in treating this cartilage injury are to achieve anatomical reduction of the osteochondral fracture, stabilize the articular cartilage surface, restore the lower limb force line, and reestablish joint stability, meanwhile minimizing surgical complications to the greatest extent possible (Joseph et al., 2019). It has been found that there is a significant cellular response to traumatic cartilage injury, which involves synoviocytes, chondrocytes and osteocytes in and around the injured joint. If these reactions are not controlled, they may lead to the development of post-traumatic osteoarthritis (PTOA) (Dilley et al., 2023). The previous view was that if the joint congruence can be achieved after intra-articular fracture and the joint stability can be achieved after ligament injury, it should have a good recovery effect. However, the current study shows that such treatment still cannot achieve the expected effect. In order to predictably and successfully treat articular cartilage injuries, it is not enough to simply restore joint congruence, limb alignment and joint stability, we must also recognize and try to mitigate the associated cellular response and promote regeneration of articular hyaline cartilage. As a result, the alternative treatments for cartilage regeneration are receiving increasing attention from joint surgeons as well as researchers.

Of course, no matter which surgical treatment option is chosen, they have a number of disadvantages, such as producing inferior fibrocartilage, causing adverse reactions at the extraction site (including the formation of cysts and intra-lesional bony outgrowths), limited availability of tissue at the extraction site, loss of phenotype due to primary chondrocyte dedifferentiation during expansion period, the possibility of requiring a second open surgery to regenerate fibrocartilage, and the immune rejection associated with allogeneic osteochondral transplantation (Behery et al., 2014). Meanwhile, compared with FTCDs and OCDs, PTCDs can prevent the adhesion of newborn chondrocytes due to its lack of blood supply and bone marrow exudate, as well as the lack of proteoglycans and chondroitin sulfate on the surface of the injured cartilage, thus resulting in the irreparable nature of partial injured cartilage. Therefore, these unique features of PTCDs also require new therapeutic approaches, and cell regenerative therapy for cartilage injury repair remain a focus of attention (Sun et al., 2022; Li H. et al., 2021).

## 4 MSC-based therapies for cartilage injury

MSCs are a special type of cells that have the ability of self-renewal in addition to differentiation, and are a class of pluripotent adult stem cells, which are widely distributed and can be isolated from a wide variety of tissues, such as bone marrow, skeletal muscle tissues, synovial membranes, periodontal ligaments, Wharton’s jelly, umbilical cords, amniotic fluids, placenta, and adipose tissues (Lou et al., 2021; Gou et al., 2024). It takes more than 50 years from discovery of MSCs to its continuous specification, definition and application. In 1968, Prof. Friedenstein et al. firstly confirmed the existence of MSCs in the bone marrow, and at the same time, he created the adherence method for isolating and culturing bone marrow-MSCs (BM-MSCs) *in vitro* (Friedenstein et al., 1968). In 1995, Prof. Caplan extracted, isolated and cultured BM-MSCs from the bone marrow of patients with malignant haematological diseases, and then infused them back into the patients to observe their therapeutic effects and prove the safety of these matrices, allowing BM-MSCs research to move from the laboratory to actual clinical applications (Caplan, 1995). In 1999, Prof. Pittenger et al. published an article in Science, proving for the first time that MSCs have the multidirectional differentiation ability, and can differentiate into adipocytes, osteoblasts and chondrocytes. This research result has inspired many researchers to study the differentiation potentials of MSCs, such as differentiating into hepatocytes, neuronal cells, and vascular endothelial cells (Pittenger et al., 1999). In 2002, some scientists discovered that MSCs have a strong immunosuppressive ability, and then discovered that MSCs themselves have a low immunogenicity, and even if they are used in different individuals or species, they are less likely to elicit immune responses, and their immune properties are very beneficial for treating immune-related diseases, including inhibiting rejection reactions and autoimmune diseases (Bartholomew et al., 2002). By 2006, the International Society for Cellular Therapy (ISCT) standardized the definition of MSCs, which can only be called MSCs if they simultaneously meet three criteria as follows: 1) the cells grow while adhering to the culture vessel.; 2) the cells can express specific antigens on their surface (markers: positive CD105, CD73 and CD90, negative CD14, CD34, CD45 or CD11b, CD79α, CD19 and MHCI); 3) the cells have the ability to differentiate to adipocytes, osteoblasts and chondrocytes (Dominici et al., 2006). According to the different stages of development, the stem cells can be classified into embryonic and adult stem cells. According to the differentiation abilities, they can be classified into totipotent, pluripotent and monopotent stem cells (Martens et al., 2013). The differentiation potentials of stem cells are closely related to their developmental stages, with a gradual decrease from embryonic stem cells to tissue-specific stem cells, and fully differentiated adult cells do not have any differentiation potentials under natural conditions.

We already know that the intrinsic healing ability of cartilage defects is limited, and small defects can be repaired spontaneously through the production of hyaline cartilage. However, larger defects can only be repaired by the production of fibrous tissue or fibrocartilage, which is biochemically and biomechanically different from normal hyaline cartilage. Some researchers have conducted in-depth studies on the therapeutic effects of MSCs, and the findings show that direct injection of MSCs is easy to be accepted by patients, which has good safety and effectiveness in repairing cartilage injury (Teo et al., 2019; Gobbi and Whyte, 2019; Tsujii et al., 2020). Of course, it has also been shown that MSCs injection alone may be less effective in the treatment of OA, and the treatment of large cartilage injuries may require a combination of surgical methods and tissue engineering techniques (Zhang X. et al., 2021). In order to enhance repair strategies for cartilage injury, combining MSCs with exogenous physical or chemical stimulation, or applying gene modification techniques to regulate the phenotype of MSCs and modify specific cell lineages has also made significant progress in cartilage regeneration (Smith and Grande, 2015; Carballo-Pedrares et al., 2023). This paper reviewed the roles of MSCs from different tissues in cartilage repair (Table 1).

**TABLE 1:** Summary of studies reported the roles of MSCs from different tissues in cartilage repair.

MSCs type	*In vitro* model	*In vivo* model	Animal model species	Processing method	Effects	References
Rat BMSCs	None	MIA-induced knee OA model	Wistar rat	BMSCs were intraarticularly injected.	BMSCs can significantly downregulate the IL-1β, IL-6 and TNF-α expression levels and upregulate IL-10 and TGF-β expression levels in MIA-induced OA cartilage.	Hamdalla et al. (2022)
	Effect of Col I/II hydrogels on the differentiation ability of BMSCs	Osteochondral defect model	Rabbit	BMSCs were encapsulated in Col I/II hydrogels.	*In vitro*, Col I/II aids BMSCs chondrogenic differentiation. *In vivo*, Col I/II hydrogel-encapsulated BM-MSCs repair cartilage with similar morphology and GAGs staining to normal tissue.	Kilmer et al. (2020)
Rabbit BMSCs	None	Osteochondral defect model	Rabbit	Runx2-overexpressing BMSCs were injected into the joint cavity.	BMSCs overexpressing Runx2 can improve the repairment of knee cartilage defects.	Hu et al. (2020)
	Effect of miR-410 on proliferation, migration and differentiation of MSCs	Osteochondral defect model	Rabbit	GelMA hydrogel loaded BMSCs overexpressing miR-410.	A bioink of GelMA and miR -410 highly expressing MSCs promotes collagen fiber regeneration and has a significant cartilage repair effect in a rabbit model.	Pei et al. (2023)
hAD-MSCs	Effect of MSCs encapsulated in the mdECM derived hydrogels on biological function and chondrogenic differentiation ability of MSCs	hAD-MSCs were implanted into dorsal regions of immunocompetent CD1 mice	CD1 mouse	A biomimetic hydrogel based on predifferentiated MSC-derived ECM subcutaneously were implanted into the dorsal regions of immunocompetent CD1 mice for 4 weeks.	mdECM can induce differenciation of MSCs into chondrocytes without the aid of any cofactors, and further form hyaline cartilage-like tissues after being implanted.	Antich et al. (2021)
Goat IFP-MSCs	Effect of Wnt modulation on chondrogenic differentiation and proliferative ability of MSCs	MSCs were implanted into the dorsal flanks of nude mice	Nude mouse	MSCs were encapsulated in plasma hydrogel, then subcutaneously implanted into nude mice.	Inhibiting TGF-β induced Wnt signaling in plasma hydrogels suppresses MSC hypertrophy and enhances chondrogenesis, yielding near normal cartilage.	Mahajan et al. (2023)
Mouse AD-MSCs	IL-1β-induced chondrocyte injury	MIA-induced OA model	Mouse	AD-MSCs were encapsulated in decorin/gellan gum hydrogel.	Decorin-enriched matrix modulates autophagy signaling to enhance AD-MSCs’ anti-inflammatory phenotype in inflammation, protecting cartilage.	He et al. (2023)
hUC-MSCs	None	Patients with cartilage defects and varus malalignment	Human	hUC-MSCs implantation or arthroscopic microdrilling were combined with high tibial osteotomy.	The cartilage repaired after hUCB-MSC implantation was more transparent and harder than that repaired after microdrilling.	Jung et al. (2024)
	None	ACLT and medial meniscectomy induced OA model	SD rat	hUC-MSCs (1 × 106/knee) in 100 μL HA were injected into the articular space of both knee joints at 4 weeks after surgery.	The HA + hUC-MSC group had a more significantly increased ICRS classification score for the femoral condyle compared with both HA group and control group.	Xing et al. (2020)

BM-MSCs, the first stem cells in human history discovered by the scientists in former Soviet Union, are MSCs isolated from bone marrow aspirates or bone marrow concentrates. As the first discovered MSCs, have been widely used in preclinical experiments and clinical validation, and possess the ability to differentiate into mesoderm lineages *in vitro*. Some researchers have also found that MSCs have an effect such as “tissue memory” triggered by epigenetic factors. Under the influence of this effect, BM-MSCs are more likely to differentiate into osteoblasts and chondrocytes,and adipose tissue-derived mesenchymal stromal cells (AD-MSCs) are more likely to differentiate into adipocytes (Ménard et al., 2020). Clinical trials have shown that BM-MSCs have characteristics such as being easy to obtain from tissues *in vivo*, rapid proliferation *in vitro*, and long-term co-existence with the host, thus showing promising therapeutic effects on a wide range of orthopaedic diseases, including spinal degenerative diseases, knee osteoarthritis, and hip osteoarthritis, etc. (El-Kadiry et al., 2021; Kouroupis et al., 2020; Eder et al., 2020). Hamdalla et al. found that BM-MSCs can promote the repair of monoiodoacetate (MIA)-induced knee cartilage injury by down-regulating the expression levels of IL-1β, IL-6, TNF-α, NF-κB, iNOS, and caspase-3, and up-regulating the expression levels of IL-10, TGF-β, and Col II (Hamdalla et al., 2022). Kilmer et al. used the scaffold composed of mixed type I and II collagen hydrogels and BM-MSCs to repair the articular cartilage defect, and found that the cell morphology and GAG staining of new cartilage tissue after scaffold implantation were similar to those of normal cartilage tissue around the defect (Kilmer et al., 2020). It was considered that the BM-MSCs modified with specific genes can better promote tissue repair. Hu et al. found that BM-MSCs overexpressing Recombinant Runt Related Transcription Factor 2 (Runx2) can enhance the repair of knee cartilage defects (Hu et al., 2020). Pei et al. used the scaffold composed of mixed MSCs highly expressing microRNA-410 and GelMA hydrogel to repair the cartilage injury in rabbits, which could promote the regeneration of collagen fibres in the cartilage (Pei et al., 2023).

The AD-MSCs are more and more commonly used in cell therapy and tissue repair. AD-MSCs can be extracted from adipose tissues such as subcutaneous fat, visceral fat, and periarticular fat. The number, apoptotic tendency, and differentiation ability of AD-MSCs vary depending on different sampling sites (Pharoun et al., 2024). Compared with BM-MSCs, AD-MSCs can be obtained directly by using minimally invasive methods such as syringe liposuction, and (0.25–0.375) × 10^6^ cells per millilitre can be obtained after 4–6 days of culture in medium containing 10% FBS. In a phase II randomized clinical trial, Lee et al. evaluated the therapeutic efficacy of intra-articular injection of autologous AD-MSCs in OA patients at 6 months after AD-MSCs injection, which resulted in an improvement in the Western Ontario and McMaster Universities Osteoarthritis index (WOMAC) score compared with before treatment. In addition, MRI showed that there was no significant change in cartilage defects, whereas the cartilage defects were aggravated in the control group (Lee et al., 2019). The study by Antich et al. showed that the decellularized bioscaffolds, which are made from extracellular matrices (ECMs) secreted by human AD-MSCs, can promote the chondrogenic differentiation of stem cells and formation of fresh cartilage tissues after *in vivo* implantation (Antich et al., 2021). The study by Mahajan et al. showed that inhibiting TGF-β-induced Wnt/β-catenin signaling can suppresses the progression of infrapatellar fat pad-derived mesenchymal stem cell (IFP-MSC) hypertrophy and enhance their chondrogenesis ability in blood plasma hydrogels. Which will generate hyaline-like cartilage with minimal hypertrophy (Mahajan et al., 2023). He et al. found that decorin-enriched matrix can enhance anti-inflammatory phenotype of AD-MSCs in the inflammation microenvironment by regulating autophagy signaling pathway, thus further protecting the cartilage (He et al., 2023). Wang et al. found that AD-MSCs from hypoxic cultures can reduce the cartilage injury, and they compared hypoxia-pretreated AD-MSCs with BM-MSCs and found that the cartilage particles differentiated from *in vitro* hypoxic BM-MSCs have a smaller particle size and a significantly higher expression level of cartilage markers compared with hypoxic AD-MSCs. Generally, there is no significant difference in the treatment of cartilage injury *in vivo* between hypoxic AD-MSCs and BM-MSCs (Wang J. P. et al., 2021). AD-MSCs are one of the best options for preclinical studies due to their easy availability and better stem cell characteristics (Strem et al., 2005).

Umbilical cord-derived MSCs (UC-MSCs) have differentiation and proliferation potentials, and are a promising candidate for use in cell therapy. UC-MSCs have a more robust gene expression profile and the ability to differentiate into other cells and are easily obtained from tissues discarded after birth, but they also bring up a number of ethical issues (Russo et al., 2022). The clinical study by Jung et al. showed that human UC-MSC (hUC-MSC) implantation has a short-term clinical effect similar to that of microdrilling as a complementary cartilage procedure in combination with high tibial osteotomy (HTO), and the cartilage repaired after hUC-MSC implantation is more transparent and harder than that repaired after microdrilling (Jung et al., 2024). Xing et al. also demonstrated that injecting UC-MSCs once a week for 6 weeks can slow down the progression of OA in rats compared with the control group when the cells are harvested after the sixth week (Xing et al., 2020). The study by Tong et al. showed that multiple administrations of UC-MSCs can slow down the progression of OA in rats by preserving the superficial cells of articular cartilage and inhibiting synovial inflammation (Tong et al., 2020). Ju et al. investigated whether there is a difference in the proliferative capacity and cartilage differentiation potential between injections of UC-MSCs and AD-MSCs in 43 OA rats, and one or two injections of AD-MSCs and UC-MSCs can significantly slow down OA progression, with significantly inhibited ECMs. Meanwhile UC-MSCs have a stronger proliferation capacity than AD-MSCs *in vitro*, indicating that researchers can collect more MSCs for cartilage injury repair in a shorter period of time, which has an important guiding significance for the clinical application of MSCs in treating cartilage injuries (Ju et al., 2022).

Synovial Membrane -derived MSCs (SM-MSCs) have gained increasing attention in recent years. These cells can be isolated from the synovial tissue of joints and possess strong proliferative and differentiation potentials, particularly in cartilage repair and regeneration (Rahmadian et al., 2024). Compared to BM-MSCs and AD-MSCs, SM-MSCs exhibit a higher capacity for chondrogenic differentiation and are more effective in generating hyaline cartilage during cartilage defect repair. Studies have shown that SM-MSCs have a higher survival rate *in vivo* and can rapidly migrate to the injury site after cartilage damage, promoting cartilage regeneration and repair (Jeyaraman et al., 2022). Furthermore, SM-MSCs are relatively easy to obtain, with a less invasive collection process and minimal ethical concerns, making them an ideal cell therapy source for clinical treatment of cartilage injuries. SM-MSCs not only exhibit strong proliferative and chondrogenic differentiation potential but also secrete various cytokines to modulate immune responses, reduce inflammation, and further enhance cartilage repair. For instance, research has shown that SM-MSCs can alleviate joint inflammation and promote cartilage repair in rheumatoid arthritis models (Li et al., 2020). These findings suggest that SM-MSCs hold great promise for cartilage repair and are worth further exploration.

MSCs can be extracted from healthy donors, and a large number of studies have reported that MSCs have a therapeutic effect in cartilage repair. However, the standardization and optimization of MSCs therapies still faces multiple obstacles: the tissue origins of MSCs affect their effects. Although multiple MSCs express similar surface markers, their immunophenotypes are different, leading to different therapeutic effects of MSCs on cartilage injuries; a lower survival rate and a higher aging rate lead to reduced economic benefit of *in vitro* proliferation, increased likelihood of cell contamination, increased difficulty in storage, and problems in maintaining optimal cell potency and viability during final delivery to patients.

## 5 MSC-EVs as a treatment for cartilage injury

### 5.1 Overview of MSC-EVs

The efficacy of MSCs is initially based on their differentiation potential to produce many different cell types that replace lost and necrotic cells in injured or diseased tissue. As research has progressed, the efficacy of MSCs, initially based on their differentiation potential, has been increasingly attributed to the fact that these cells promote tissue regeneration and repair through the release of secretory factors. These secretory factors, collectively known as secretome, consist of soluble proteins, free nucleic acids, and lipids (Vizoso et al., 2017; Théry et al., 2002). The secretome was originally proposed by Tjalsma et al. in 2004, they used it as a general term to describe all secretory proteins and secretory mechanisms of cells when studying the roles of secretory proteins in supporting bacterial survival (Tjalsma et al., 2000). Subsequently, this definition was developed by Hathout and Agrawal with a more detailed concept. The secretomes are the factors secreted into the extracellular space by cells, tissues or organisms under a certain condition and within a certain time frame (Hathout, 2007; Agrawal et al., 2010). Currently, this definition has been further updated, and the secretomes contain EVs with important molecules in addition to known soluble factors and lipids. More and more studies have pointed out that the secretomes are an important mediator in promoting tissue regeneration, with a potential to regulate cell signal transduction and promote tissue repair (Phinney and Pittenger, 2017; Lai et al., 2015; Gomes et al., 2018). The following sections will explore in depth the basic characteristics of MSC derived extracellular vesicles (MSC-EVs) and its application in cartilage injury repair.

EVs are nano-to micrometre-sized natural membrane vesicles encapsulated by a phospholipid bilayer, with a size of 50–150 nm, and a protein density of 1.1–1.18 g/mL. However, it has also been reported that the diameters of EVs are 50–200 nm, and EVs contain endosomal-related proteins such as TSG101 and ALIX, as well as tetraspanin superfamily proteins such as CD9, CD63 and CD81 (Théry et al., 2006).

Almost all cells can secrete EVs, which are released into the extracellular space in an active or inductive manner (Cheng and Hill, 2022). Consistent with their intracellular origin, the lipid membranes of EVs are enriched in cholesterol, sphingomyelin, and ceramides, which are typical lipid-rich membranes, and can reflect the metabolic state of the body and the functions of the progenitor cells under different pathological conditions (Wu et al., 2021; Cicero et al., 2015; Simons and Raposo, 2009).

Meanwhile, EVs participate in physiological activities such as immune responses, antigen presentation, organ development, and reproduction processes in the body. In particular, EVs derived from stem cells, have been shown to be effective in the treatment of ischemic stroke (IS), spinal cord injury (SCI), OA and many other types of diseases (Zhang Z. et al., 2021; Wang Y. et al., 2021; Yin et al., 2022). Recent studies have also shown that EVs have an important role in regulating intercellular communication. When EVs are internalized, the receptor cells respond to the EV-loaded molecules and genes, further affecting and changing the function of the receptor cells (Kalluri and LeBleu, 2020).

In the field of cartilage injury repair, MSC-EVs represent a potentially innovative therapeutic approach. They promote cartilage tissue repair by regulating the proliferation and differentiation of chondrocytes and synthesizing collagen matrix (Contentin et al., 2022; Chen et al., 2019; Chen et al., 2018; Wu et al., 2019). Meanwhile, another outstanding advantage of MSC-EVs is that they can be used as carriers to carry drugs, and various engineering techniques can be used to load the target substances into vesicles to transport them to specific target cells. In the following, we refer to and summarize the existing research findings, and evaluate the therapeutic efficacy of MSC-EVs in treating articular cartilage injuries (Table 2).

**TABLE 2:** Summary of animal studies reported the roles of MSC-EVs in cartilage repair.

MSC-EVs type	*In vitro* model	*In vivo* model	Animal model species	Processing method	Effects	References
Equine BMSC -EVs	eACs were co-cultured with EVs	None	None	The expression of healthy cartilage/OA and proliferation markers was evaluated in eACs (monolayers or organoids).	Compared with the equine BM-MSCs, equine BM-MSC-EVs can affect the phenotype of eACs more obviously and increase the expression of chondrocyte functional markers and cell migration more effectively, thus potentially slowing the progression of OA.	Contentin et al. (2022)
BMSC -EXOs	The chondrocytes isolated from the OA rats were co-cultured with EXOs	ACLT- induced OA model	Rat	BMSC-EXOs after LIPUS stimulation can exert an effect on the biological function of OA chondrocytes *in vitro* and a protective effect on cartilage injury *in vivo*.	LIPUS can enhances the repair effect of MSCs on OA cartilage, and its underlying mechanism is related to increased autophagy-mediated exosome release.	Xia et al. (2022)
UC-MSCs-EXOs	Chondrocytes were co-cultured with EXOs	Cartilage defect model	Rat	Effect of UC-MSCs-EXOs in mechanical environment of RCCS on the biological function of chondrocytes was investigated.	The mechanical stimulation can increase the yield of exosomes and its biological function in the repair of cartilage defects.	Yan et al. (2020)
hAD-MSCs-EVs	Hypoxia-preconditioned AD-MSCs-EVs co-cultured with BM-MSCs and chondrocytes, respectively	Cartilage defect model	Rat	Investigation of whether hypoxia preconditioned AD-MSCs-EVs affect BM-MSCs and chondrocytes *in vitro* and cartilage repair *in vivo* compared to normal.	A modified gelatin matrix/3D-printed ECM scaffold - based ApoEVs delivery system with hypoxic preconditioning boosts MSC - ApoEVs’ function and cartilage repair.	Ding et al. (2024)
hSM-MSC-EVs	IL-1β induced OA SW1353 were co-cultured with EVs	Both the medial collateral ligament and the medial meniscus were completely transected	Rat	Effects of SM-MSC-EVs containing miR-26a-5p on biological function of OA SW1353, inflammation *in vitro* and their action mechanisms and repair effect on OA were explored *in vitro* and *in vivo*.	SM-MSC-EVs can transfer miR-26a-5p into chondrocytes to upregulate miR-26a-5p and inhibit PTEN, thereby inhibiting apoptosis and inflammation and ameliorating cartilage injury in OA.	Lu et al. (2021b)
hMSCs -EXOs	OA chondrocytes induced by IL-1β was co-cultured with EXOs	DMM and ACLT- induced OA model	Rat	The *in vitro* and *in vivo* exploration of hMSCs-EXOs with miR-199a-3p on OA chondrocytes’ function, mechanism, and repair effect.	hMSCs-EXOs can partially alleviate the pathological severity degree through the miR-199a-3p-mediated mTOR-autophagy pathway in animal OA model.	Zhao et al. (2023)
hUMSC -EXOs	EVs were co-cultured with BMSCs, chondrocytes and macrophages	Osteochondral defect model	Rabbit & Rat	Weather MSC-EXOs can enhance the reparative effect of ACECM scaffold and its underlying mechanism were explored.	In rabbit and rat models, hWJ-MSC-Exos enhance ACECM scaffold effect and promote osteochondral regeneration and regulate joint microenvironment.	Jiang et al. (2021)
BMSCs -EVs	Chondrogenic potentials and matrix formation of EVs derived respectively from naïve MSC, chondrogenically primed MSCs, chondrocytes, and co-cultures of chondrocytes plus MSCs at different ratios were evaluated *in vitro*	MIA-induced OA model	Rat	The chondrogenic potential of the EVs was investigated.	EVs derived from a higher ratio of chondrocytes to BM-MSCs have a better chondrogenic effect in the treatment of osteochondritis.	Hosseinzadeh et al. (2023)
hWJ-MSC-EVs	hBM-MSCs and chondrocytes were co-cultured with EVs	Osteochondral defect model	Rabbit	*In vitro*, hWJ-MSC-EVs co-cultured with hBM-MSCs and chondrocytes to observe effects. *In vivo*, related mechanism and repair effect on osteochondral defect were explored.	hWJ-MSC-EVs can promote cartilage regeneration and repair via the microfracture-mediated ITGB1/TGF-β/Smad2/3 axis.	Chen et al. (2024)

### 5.2 Sources of MSC-EVs

All cells, both prokaryotes and eukaryotes, release EVs. EVs can be broadly classified into two categories, ectosomes and exosomes. Ectosomes are EVs produced by directly budding outwards from the plasma membrane, including microvesicles, microparticles, and large vesicles with a diameter of approximately 30 nm to 1 mm. Exosomes are EVs with a diameter of 30–200 nm (an average of about 100 nm), they originate from endosomes, and are formed by the release of intracellular endosomes into the extracellular space through cytosolisation. Continuous invaginations of the plasma membrane eventually lead to the formation of multivesicular vesicles, which can intersect with other vesicles and organelles within the cell, resulting in a diversity in exosome components. Depending on the cell of origin, EVs, including exosomes, may contain many cellular components, such as DNA, RNA, lipids, metabolites, and cytoplasmic and cell surface proteins (Gurung et al., 2021). The physiological purpose for producing EVs remains largely unknown. Some studies have suggested that EVs are considered as metabolic waste products that may remove excess and/or unnecessary components from the cells to maintain a stable intracellular environment, and some studies also believe that EVs can be used as a transmitter of genetic information, which can carry and transfer their own signal molecules to nearby or even distant cells, and further regulate the physiological and pathological states of the recipient cells, and are involved in the occurrence and development of various diseases (Van Niel et al., 2018).

MSC-EVs are isolated from a variety of body fluids, including plasma, breast milk, ascites, urine, saliva, bone marrow, infrapatellar fat pads, synovium, and human embryonic tissues, etc. They may represent the states of their donor cells, and play an important role in mediating cellular communication (Cicero et al., 2015; Chen et al., 2019; Chen et al., 2018; Van Niel et al., 2018; Bobis-Wozowicz et al., 2017; Yáñez-Mó et al., 2015; Cosenza et al., 2017; Mao et al., 2018). MSCs are very sensitive to environmental changes and show different secretion profiles and phenotypes under different stimulus conditions. Hypoxia, mechanical environment, and proinflammatory stimulation can induce MSCs to secrete more EVs with a higher therapeutic efficacy, and MSCs can also be genetically edited to upregulate the expressions of some RNAs or proteins in the MSC-EVs, which can promote tissue repair. Xia et al. found that the low-intensity pulsed ultrasound can promote the effect of BM-MSCs in cartilage repair in patients with osteoarthritis by regulating the release of exosomes mediated by autophagy (Xia et al., 2022). Yan et al. found that the exosomes derived from UC-MSCs can improved osteochondral activity by upregulating the expression level of lncRNA H19 in a mechanical environment (Yan et al., 2020). Ding et al. found that a delivery system for apoptotic extracellular vesicles (ApoEVs) based on a modified gelatine matrix/3D-printed ECM scaffold and hypoxic preconditioning can improve the functionality of stem cell-derived ApoEVs, and also facilitate the repair of cartilage injury (Ding et al., 2024). Lu et al. reported that SM-MSC-EVs over-expressing miR-26a-5p can better repair OA cartilage injury by inhibiting apoptosis and inflammatory responses (Lu L. et al., 2021). There are also articles that compare different MSC-EVs. Zhu et al. compared iPSCs -EVs with SM-MSC-EVs, indicating that there are relatively less S-O staining of articular cartilage after treatment with iPSCs -EVs, suggesting that the content of GAGs deposited in cartilage is low (Zhu et al., 2017). However, all these specific MSC-EVs or modified MSC-EVs are investigated in single studies. All the conclusions have not been confirmed by two or more studies. There is still no clear recommendation as to whether there is a certain type of MSC-EVs that has the best protective effect on the articular cartilage.

### 5.3 Extraction of MSC-EVs

EVs can be extracted by a variety of methods, and according to a survey conducted by the International Society for Extracellular Vesicles (ISEV), the gradient ultracentrifugation method is by far the most widely used method for separating EVs, and it is also considered as the “gold standard” for EVs extraction (Gardiner et al., 2016). With the rapid advancement of technology, some researchers have developed new methods to efficiently separate EVs from complex biological fluids, including not only microfluidic separation techniques (Gholizadeh et al., 2017), commercial kits based on acoustics (Wu et al., 2017), electrophoresis (Ibsen et al., 2017), deterministic lateral displacement pillar arrays (Wunsch et al., 2016), viscoelastic microfluidic system (Liu C. et al., 2017; Zhou et al., 2019) and immunoaffinity (Hisey et al., 2018), and the asymmetric flow field-flow fractionation techniques (Zhang and Lyden, 2019), etc., but also emerging nanomaterial separation methods such as magnetic nanowires (Lim et al., 2019). Purification of specific subpopulations of EVs remains difficult due to overlap in size, similarity in composition and lack of specific markers. Furthermore, currently EVs are largely heterogeneous. In light of this, ISEV recommends the use of MSC-EVs for the description of EVs without the need for further proof of their origins (Théry et al., 2018). In addition, ISEV also encourages relevant authors to describe the size, biochemical composition and functional location of EVs when conducting research on them.

Extraction of MSC-EVs is a very important step in research, which affects its study in various fields. Different extraction methods have their own advantages and disadvantages, and need to be selected according to the study objectives and requirements. Meanwhile, in view of the fact that the preparation methods and storage conditions of MSC-EVs may affect the therapeutic effects, there is a greater need for quality control and standardized preparation of MSC-EVs, thus ensuring the consistency of therapeutic effects and meeting the requirements of drug regulatory agencies. It is needed to further define the biological properties, preparation methods and purification techniques of MSC-EVs in the future studies.

### 5.4 Identification of MSC-EVs

EVs have a characteristic morphology on their surfaces, and their inner parts contain biomolecules such as proteins, lipids, and nucleic acids, and measurements of these components can be used as an alternative to quantifying EVs. However, these parameters are exactly equivalent to the actual parameters of EVs. Typically EVs are quantified using one or more of the following indicators: particle number, total protein content, total lipid abundance, total RNA, or specific molecules.

EVs can be counted and analyzed using the light scattering techniques (LST) (Bağcı et al., 2022), nanoparticle tracking analysis (NTA) (Auger et al., 2022), flow cytometry experiments (Welsh et al., 2020), resistive-pulse sensing (for various sizes of EVs depending on the aperture of the sensor) (Cimorelli et al., 2021), scanning electron microscope (SEM) (Cizmar and Yuana, 2017), atomic force microscope (AFM) (Skliar and Chernyshev, 2019), or a detection platform that combine the surface plasmon resonance (SPR) technology and AFM (Chin et al., 2020). Because the accuracy of the applied method has strong requirements on the experimental platform and the purity of the sample, the number of particles can only be accurately quantified within a certain range of particle concentration and particle size. In addition, the particle counting technology may have a bias in a specific range of particle concentration and size due to its lower sensitivity to smaller particles. The total protein content of EVs can be determined by standard colourimetric methods, fluorometric methods, or the methods of protein bands staining on SDS-PAGE (Théry et al., 2018). Overestimation in EVs quantification due to the presence of co-isolated protein contaminants is a major drawback of this method. The sulfo-phospho-vanillin method is adopted to quantify total lipid by using EV lipid bilayers containing fluorescent phospholipid dyes (Osteikoetxea et al., 2017; Benmoussa et al., 2017) or fourier transform attenuated total reflection infrared spectroscopy (ATR-FTIR) (Mihály et al., 2017). However, these techniques either require specialized equipment or may be insufficiently sensitive for detection of small amounts of EVs. Meanwhile, not all EVs can be detected due to the difference in composition among various lipids. Other identification methods include total RNA quantification, and certain specific EVs can be determined using a colorimetric analysis based on aptamers and carbon nanotube (Xia et al., 2017).

Different MSC-EV identification methods have their own advantages and disadvantages. For example, the transmission electron microscopy (TEM) has a high resolution, but requires an expensive instrument and specialized skills. The mass spectrometry can provide detailed molecular information, but requires complex sample preparation. The flow cytometry can perform high-throughput analysis, but has limited resolution for small-sized EVs. The identification of MSC-EVs is a critical step in medical research, and the researchers need to use a variety of identification methods to gain a deeper understanding of their properties and applications. Different identification methods have their own advantages and challenges, and these methods can be used in combination or selected based on the research objectives and requirements.

### 5.5 Administration method of MSC-EVs

EVs are membranous natural nanoparticles that are released by all cell types, and their inner parts contain active biomolecules produced by them, which are transferred between cells. EVs have multiple inherent characteristics such as immunological tolerance, stability in circulation system and high biobarrier penetration ability to reach distant organs such as brain. These characteristics make EVs an excellent nanocarrier for the future treatment of diseases. The technologies used to load drugs or materials (which we also call cargo) into EVs are broadly divided into two categories: exogenous loading and endogenous loading (Lu et al., 2024). The exogenous loading typically involves loading the cargo into prepared EVs using a variety of methods, including co-incubation, ultrasonic treatment, and electroporation (Komuro et al., 2022). Shu Zhao et al. transferred miR-199a-3p into the exosomes of subcutaneous adipose stem cells by electrotransfer, so that the exosomes could enter chondrocytes and deep joint tissues *in vivo* as engineered exosomes *in vitro*, and it was found that the exosomes have a good protective effect on injured cartilage (Zhao et al., 2023). To perform endogenous loading, EVs-secreting cells are genetically engineered to overexpress the required RNAs or proteins, and modify them with or without a specific way, which are then absorbed by the target cells during the biological transport processes of EVs (Armstrong et al., 2017; Corso et al., 2019; Zickler and El Andaloussi, 2020).

Currently there are two main ways of administration of EVs for the treatment of articular cartilage damage: intra-articular injection; mixing with materials such as biological materials (Li et al., 2019). MSC-EVs are injected directly into the damaged cartilage area. The advantage is that MSC-EVs can act directly on the damaged area to accelerate the repair process, and it is low invasive and does not require open surgery, reducing the risk of surgery for patients (Kodama et al., 2022). However, the disadvantage of direct injection is that it is difficult to ensure that MSC-EVs are uniformly distributed throughout the damaged area, and MSC-EVs may be rapidly cleared by the body, which affects their therapeutic effects. Other studies have also reported the advantages of combining MSC-EVs with stents, MSC-EVs are used in combination with biological materials, which are implanted into the injured area. For example, Liu X. et al. reported that EVs implanted in hydrogel patches are superior to single injections of EVs (Liu X. et al., 2017). Shuangpeng Jiang et al. found that the exosomes originating from human umbilical cord Wharton’s jelly-derived mesenchymal stem cells (hWJ-MSC-Exos) can enhance the effect of ACECM scaffold and promote the osteochondral regeneration (Jiang et al., 2021). The advantages of this approach are that the carrier and the bioscaffold can control the release rate of MSC-EVs, prolong the therapeutic effect and have better stability, this approach compensates for the shortcomings of direct articular injections and demonstrates a synergistic enhancement of cartilage repair. Of course, this scaffold approach is also surgically invasive, increasing the risk of surgery and the possibility of prognostic infection. In addition, some soluble carriers may dissolve *in vivo*, leading to failure of MSC-EVs therapy.

Regarding the *in vivo* dosage of EVs, there is still a lack of studies that have comprehensively assessed the *in vivo* dose-response kinetics. Most studies quantify the *in vivo* applied dose of EVs based on the total protein amount (Gao et al., 2020), while other methods such as the use of NTA to measure the particle number of vesicles are used as the basis for EVs dose quantification (Comfort et al., 2021). Furthermore, in terms of investigating whether the variation in the dose of different EVs is mainly due to differences in animal species, it has also been speculated that higher animal species tend to require lower doses to observe similar therapeutic effects in larger animal species due to their lower metabolic rates and body surface area to weight ratios (Nair and Jacob, 2016).

In summary, in future research, the combination of MSC-EVs with patient-specific cartilage pathology can be considered for personalized treatment. In terms of biocarrier research, we can focus on developing more stable carriers and scaffolds to improve the release efficiency and treatment duration of MSC-EVs. More clinical trials will help to validate the safety and efficacy of different modes of administration and promote the clinical application of MSC-EVs. The mode of administration of MSC-EVs, as a new approach to treating cartilage injury, is crucial. Different modes of administration have their own advantages and challenges, and future studies will help optimize these methods and improve the efficacy of MSC-EVs treatment.

### 5.6 Animal research models for MSC-EVs

Despite the existence of guidelines for *in vivo* animal experiments, the methodology of animal experiments can still be further improved in many studies (Hooijmans et al., 2014; Kilkenny et al., 2010). Mice are the most commonly used experimental animals, and MSC-EVs have been widely used in mouse models. Kendrick TO et al. used mice to prepare three cartilage injury models including DMM, cartilage defects and collagenase erosion and used the human MSC-EVs in the treatment of cartilage injury, and found that transplantation of MSC-EVs into the injured cartilage can effectively reduce cartilage loss in a mouse model of cartilage injury (To et al., 2020). Zhao et al. intravenously injected MSC-EVs in mouse models, and found that MSC-EVs can attenuate mitochondrial damage and inflammation by stabilizing mitochondrial DNA (Zhao et al., 2021). The study of Duan et al. on the surgical destabilization of the medial meniscus (DMM) model of osteoarthritis in mice showed that EVs derived from LPS-pretreated human synovial MSC-EVs can inhibit ECM degradation and prevent knee OA (Duan et al., 2021). Rat models are also commonly used in the studies on MSC-EVs. Hosseinzadeh et al. found that in a rat model of osteoarthritis, a higher ratio of chondrocytes/MSCs can improve the therapeutic efficacy of EVs harvested from chondrocyte/MSC co-cultures, thus repairing cartilage injury (Hosseinzadeh et al., 2023). Yang et al. compared the paracrine effects of secretomes between aerobically and hypoxia pretreated MSCs in a rat osteochondral defect model, and demonstrated that a relatively low dose of hypoxia-conditioned medium and corresponding EVs can effectively promote the repair of osteochondral defects and attenuate the joint inflammation in a rat osteochondral defect model (Yang et al., 2023). The study on an osteochondral defect model by Zhian Chen et al. indicated that hWJ-MSC-EVs can promote cartilage regeneration and repair via the microfracture-mediated ITGB1/TGF-β/Smad2/3 axis (Chen et al., 2024). Pigs are one of the important animal models used in surgery and disease research, and the researchers have used pig models to investigate the application of EVs in cardiac surgery, organ transplantation and tissue regeneration engineering. For example, Shipin Zhang et al. found that the combination of MSC exosomes and HA, administered at a clinically acceptable frequency of three weekly intra-articular injections, can promote functional cartilage and subchondral bone repair (Zhang SP. et al., 2022). Hede KTC et al. created a model of cartilage defects in the knee joint in a minipig and found that MSC-EVs can promote cartilage regeneration (Hede et al., 2021).

Describing the pain and disability in animal subjects is a huge challenge, and there is currently no gold standard for validating methods of pain measurement, gait analysis and standardized functional assessment in OA animal model. The changes in gait have been used in attempts to validate pain and disability (Shah et al., 2020), but it is important to note that changes in gait may be the result of pain-related avoidance or biomechanical changes caused by joint dysfunction. Linking the study finding of advanced imaging techniques with the indicators of OA progression, in combination with measurements of histology, cartilage mechanical properties, and gait function, is an important direction for future studies on cartilage injury. To date, there is no gold standard for biomarker detection or imaging used in animal cartilage injury.

These animal models provide good opportunities for researchers to better understand the functions of MSC-EVs in biology and medicine and their potential applications in cartilage repair. Although all current research methods seem reasonable, there is a lack of detailed data on experimental animal parameters in the articles. Meanwhile, current studies are still largely limited to small animal studies and further efforts should be made to progress this research to large animal studies and eventually to clinical studies. Similarly, safety and regulatory issues must be addressed before MSC-EVs can be used in clinical therapy. This includes determining the optimal preparation method, dosage, route and storage conditions for MSC-EVs, as well as monitoring and reporting potential adverse events. This emphasizes the importance of adhering to laboratory animal guidelines and methods in future publications to enhance the credibility and reliability of future articles. In addition, more animal studies and clinical trials will help validate their benefits in the treatment of different diseases and promote their application in clinical practice.

## 6 Effect of MSC-EVs on cartilage injury

### 6.1 MSC-EVs promote the vitality, proliferation, and migration of chondrocytes

It is well known that MSC-EVs carry a variety of proteins and growth factors, such as transforming growth factor β (TGF-β), type II collagen, and BMP-2. These biomolecules play key roles in cartilage injury repair. Yoo et al. demonstrated that TGF-β is a growth factor that plays a key role in chondrocyte proliferation and collagen matrix synthesis, and described the important effect of the TGF-β family on OA and the possibility of treating OA with MSC-Exos (Yoo et al., 2022). Chen et al. found that hWJ-MSC-EVs can carry integrin beta-1 (ITGB1), and the cartilage will overexpress ITGB1 after EVs uptake, thereby activating the TGF-β/Smad2/3 axis and improving the activity and proliferation ability of chondrocytes (Chen et al., 2024). MSC-EVs can carry abundant miRNAs and LncRNAs such as miRNA-18-3p, miR-140, miR-135b, miR-181c-5p, and miR-21-5p, which will also play a huge role in promoting chondrocyte proliferation and migration through regulating key genes and cell proliferation signalling pathways. These related signalling pathways include Wnt/β-catenin, PI3K/Akt, MAPK, and ERK signalling pathways, etc., which can stimulate the division and proliferation of chondrocytes. Zhang B. et al. found that hypoxia-pretreated MSCs can secrete EVs, which may stimulate chondrocyte proliferation and migration through the miRNA-18-3p/JAK/STAT or miRNA-181c-5p/MAPK signaling pathways, thereby promoting cartilage repair (Zhang B. et al., 2022). Wang et al. demonstrated that TGF-β1 stimulation can enhance miR-135b expression in MSC-Exos, and MSC-Exos-derived miR-135b can increase the viability of chondrocytes and promote the proliferation of chondrocytes, and ultimately promote cartilage repair (Wang et al., 2018). Wan S. et al. investigated the urine-derived stem cell extracellular vesicles (USCs-EVs) and pretreated them with hypoxia, and found that EVs can enhance chondrocyte proliferation and migration by providing miR-26a-5p, demonstrating that the effect of EVs-miR-26a-5p in promoting chondrocyte proliferation and migration is mediated by its regulation of PTEN (Zhou et al., 2022). Zhang Q. et al. investigated and emphasized the repairing effect of hUC-MSC-EVs carrying miR-181c-5p on cartilage injury, indicating that miR-181c-5p can exert a targeted inhibition effect on the expression of SMAD7 to promote the proliferation, migration, and chondrogenic potential of BM-MSCs induced by BMP-2 (Zhang Q. et al., 2022).

Cartilage tissue is a key component of the skeletal system and has important functions in supporting, cushioning and protecting joints. However, the cartilage injury and degenerative diseases often lead to cartilage degeneration, which can have a significant impact on patients’ quality of life. Understanding the mechanisms of chondrocyte viability, proliferation and migration is critical for understanding the processes underlying cartilage maintenance and repair. Therefore, it is of great clinical value to find effective methods to promote cartilage vitality, proliferation and migration to achieve cartilage repair and regeneration.

### 6.2 MSC-EVs promote matrix synthesis

Chondrocytes are the main cell type in cartilage tissue, and they are responsible for synthesizing and maintaining the cartilage matrix such as collagen, proteoglycans, and other molecules, which determine the strength and elasticity of the cartilage. The traditional treatments are to alleviate symptoms rather than to actually promote cartilage repair. One of the key steps in cartilage repair is the synthesis of a new collagen matrix, which helps to maintain the structure and stability of cartilage tissue. Hao et al. found that in a nucleus pulposus cell (NPC) apoptosis model induced by tumor necrosis factor-α (TNF-α), miR-217 expression was reduced, and subsequently miR-217 expression was increased by transferring miR-217 from MSC-EVs to NPCs, thereby weakening NPC apoptosis and ECM degradation (Col II and aggrecan were elevated mainly by decreasing the expressions of MMP13 and ADAMTS5) (Hao et al., 2022). Zhou et al. found that hUC-MSCs -EVs can decrease the m6A level of NLRP3 mRNA with miR-1208 targeting combined with METTL3, inhibit the secretion of pro-inflammatory factors and the degradation of cartilage ECM, and thus alleviate the progression of OA in mice. And provide a new approach for clinical treatment of OA (Zhou et al., 2022). Woo et al. investigated the therapeutic potential of hAD-MSC-EVs in alleviating OA and its mechanism, they used a mono sodium iodoacetate (MIA) to establish an arthritis model and a destabilization of medial meniscus (DMM) mouse model, and the intra-articular injections of hADSC-EVs can promote the production of Col II, while inhibiting the expressions of MMP-1, MMP-3, MMP-13 and ADAMTS-5 (Woo et al., 2020). These studies suggest that MSC-EVs can not only play a role in promoting matrix synthesis such as Col II, but also simultaneously weaken the effects of matrix-degrading enzymes such as MMP13 and ADAMTS-5. Interestingly, Hosseinzadeh et al. found that EVs isolated from the co-culture of a high proportion of chondrocytes and MSCs can promote chondrogenesis and facilitate cartilage repair in rat OA models (Hosseinzadeh et al., 2023). The degenerative diseases of cartilage can disrupt these biological processes, leading to the degradation of cartilage. Therefore, matrix synthesis and degradation are essential for cartilage regeneration. MSC-EVs can stimulate chondrocyte synthesis of collagen and other matrix molecules, thereby helping to repair the injured cartilage tissue.

### 6.3 MSC-EVs participate in immune regulation

Inflammatory infiltration of synovial membrane by immune cells such as macrophages, T cells and B cells plays a key role in the pathogenesis of degenerative cartilage disease or cartilage injury. Therefore, modulating the local inflammatory microenvironment and tissue regenerative microenvironment is important for the treatment of cartilage injury. Paracrine effects mediated by MSC-EVs have recently been considered as one of mechanisms for their therapeutic properties. MSC-EVs carry a variety of growth factors, chemokines and other signaling molecules that affect the polarization, maturation, proliferation and migration of macrophages, and recent studies have demonstrated that MSC-EVs can regulate the polarization of macrophages by inducing the conversion of the M1 phenotype to the M2 phenotype, thereby promoting the healing process (Zhao et al., 2020). Zhang et al. found that MSC-EVs have an immunomodulatory effect, which can attract M2 macrophages to infiltrate the OA cartilage defect and synovium, reduce the infiltration of M1 macrophages, downregulate the expressions of IL-1β and TNF-α, and thus inhibit the inflammatory response in OA (Zhang et al., 2018). Zha Xi et al. found that M2 macrophage derived exosomes have a therapeutic effect on knee osteoarthritis (KOA) rats by inhibiting the PI3K/Akt/mTOR signaling pathway, which can alleviate the inflammatory response and pathological damage of articular cartilage in KOA rats. In addition, after being treated with M2 macrophage-derived exosomes (M2-EXOs), the key proteins such as aggrecan, Col-10, SOX6, and Runx2 associated with KOA were significantly elevated, while MMP-13 was significantly inhibited (Da-Wa et al., 2021). Wang and Xiu found that the OA-induced M1 polarization of synovial macrophages is inhibited by BM-MSC-Exos carrying TGF-β1, and BM-MSC-Exos with TGF-β1 reduce cartilage injury in OA rats by carrying highly expressed miR-135b (Wang and Xu, 2021). The effects of MSC-EVs on T cells have been confirmed both *in vivo* and *in vitro*. For example, Chen et al. co-cultured peripheral blood mononuclear cells with MSC-EVs, and found that MSC-EVs can induce the conversion of TH1 cells to TH2 cells, reduce the potential of T cells to differentiate into TH17 cells, and increase the amount of regulatory T cells (Tregs) (Chen et al., 2016). Cosenza et al. evaluated the immunosuppressive effects of EVs on T cells in a model of delayed-type hypersensitivity reaction, and found that MSC-EVs can inhibit T cell proliferation in a dose-dependent manner and thus induce Treg populations to exert an immunomodulatory effect on inflammatory arthritis (Cosenza et al., 2018).

The effects of MSC-EVs in immune regulation can be broadly divided into the following three aspects: 1) inhibition of T-cell activity, MSC-EVs can reduce the functions of activated T-cells, decrease immune responses, and thus contribute to the treatment of immune disorders; 2) modulation of immune cell functions, MSC -EVs can affect a variety of immune cells such as B cells, natural killer cells and anti-inflammatory cells, and regulate the balance of the immune system; 3) promotion of immune tolerance, MSC-EVs can help to promote immune tolerance and reduce the occurrence and development of autoimmune diseases. Therefore, MSC-EVs have an immunophenotype that can facilitate tissue repair and regeneration due to their effective immunomodulatory properties and anti-inflammatory ability, and are widely believed to have the potential to promote cartilage regeneration.

### 6.4 MSC-EVs promote the morphological improvement of cartilage tissue

Articular cartilage is a crucial tissue in the joints, mainly consists of chondrocytes, type II collagen, proteoglycans and water, and has functions such as lubrication, shock absorption and decompression, thus playing a key role in providing cushioning and support. However, articular cartilage is affected by injury or degenerative disease, which may result in histomorphological changes. Chondrocytes are located within the cartilage lacuna and are surrounded by a rich cartilage matrix. Some studies have found that after MSC-EV treatment, the newborn cartilage has morphological features similar to those of native cartilage, and is also described as a cartilage that has completely covered the defective area and is integrated with the native cartilage (Zhang et al., 2018). In some studies, strong staining of type II collagen and GAGs and weak staining of type I collagen were observed, suggesting that the newly formed tissue resembles the hyaline native cartilage (Zhu et al., 2017). Of course, some studies have found that after MSC-EVs treatment, the newly formed cartilage did not have the morphological characteristics of native cartilage, but it was still improved compared to the phosphate buffered saline (PBS) group. Zhang et al. found that after MSC-EVs treatment, the newly formed tissues showed significant ECM staining for type VI collagen, and lubricin-positive cells were present in the superficial and mid-region of the repaired cartilage. In addition, the newly formed cartilage tissues showed a slight hypertrophy and a low level of type X collagen. In contrast, the PBS control group showed mainly fibrous tissue, with stronger staining of type I collagen, and lower staining of GAGs and type II collagen (Zhang et al., 2016).

At present, the commonly used scores for macroscopic evaluation of cartilage repair include the International Cartilage Repair Society (ICRS) classification score and Oswestry Arthroscopy Score (OAS). The ICRS classification score was designed by Peterson et al. (2000), Carballo et al. (2017), the OAS was designed by the OsCell team. In addition, in order to better assess the condition of OA patients, the Osteoarthritis Research Society International (OARSI) grading system, which is jointly developed by the European League Against Rheumatism and American College of Rheumatology, is one of the mainstream methods used in clinical practice to assess the disease severity of OA patients. RAltman et al. proposed the clinical classification criteria for osteoarthritis, laying the foundation for its subsequent development, whose work contributed to the classification and grading of knee osteoarthritis (Altman et al., 1986). A safe and reliable scoring system is essential to assess cartilage repair, which should serve as a cornerstone, demonstrating evidence of the repair effectiveness for cartilage injuries. However, until now, there has been no unified criteria for scoring the degree of cartilage repair.

## 7 Conclusions and future perspectives

**FIGURE 2:**
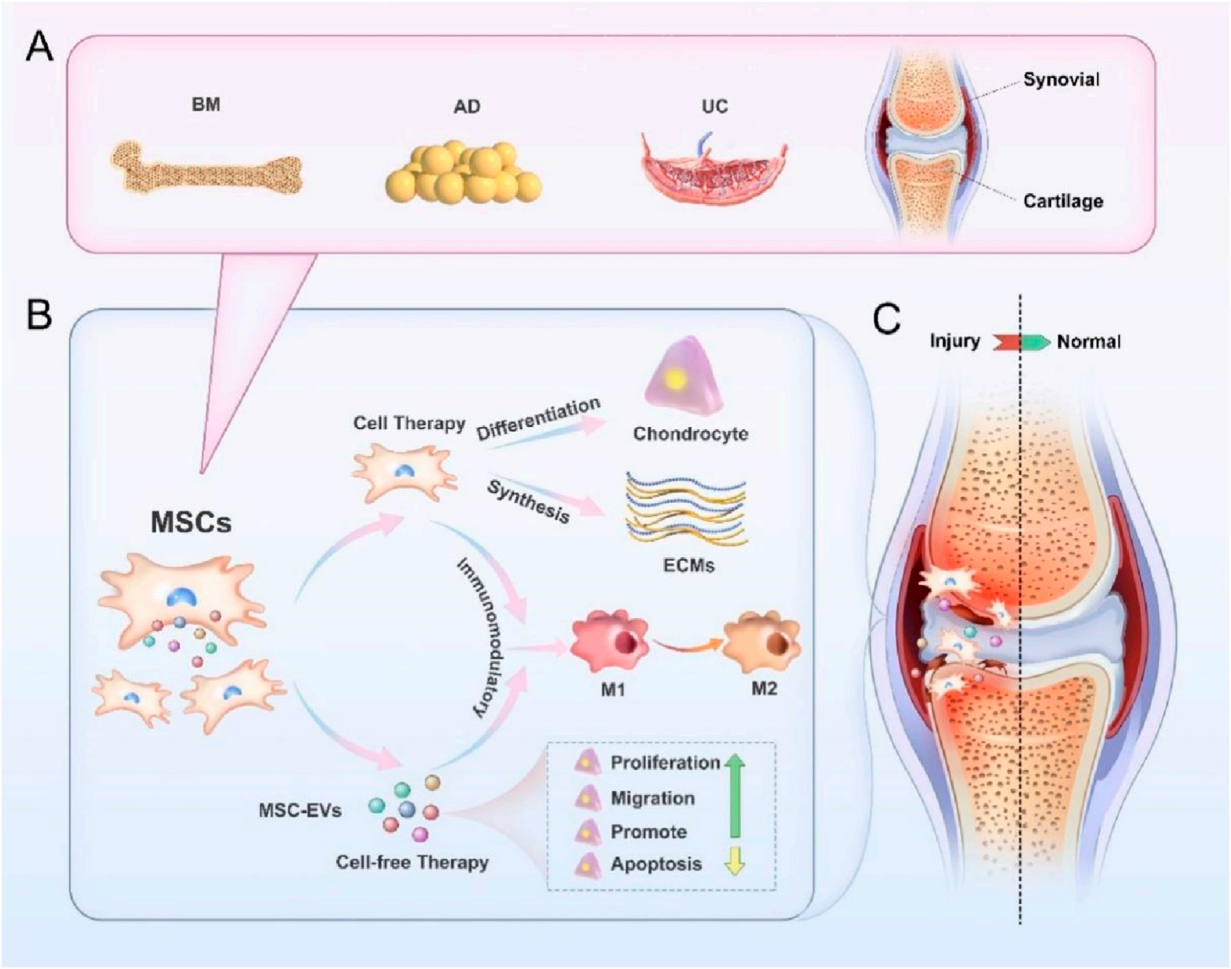
Action mechanism of MSCs and their EVs in cartilage injury repair. **(A)** Tissue sources of MSCs, including bone marrow, adipose, umbilical cord, synovium and joint, etc. **(B)** MSCs can repair cartilage injuries via cell replacement and regeneration therapy. MSCs can differentiate into chondrocytes, synthesize extracellular matrix, regulate intracellular immune environment, thus promoting macrophage polarization from M1 to M2. MSC-EVs can repair the cartilage injuries via “cell-free” replacement and regeneration therapy, inhibit the release of inflammatory factors and promote the macrophage polarization from M1 to M2, and they can also promote the proliferation, migration, matrix synthesis, and inhibit the apoptosis of chondrocytes. **(C)** MSCs and MSC-EVs can be intra-articularly injected or encapsulated in biomaterials to be implanted into the injured cartilage, which can facilitate cartilage repair, and promote the regeneration of hyaline cartilage.

In recent years, the concept of precision medicine has been receiving more and more attention because of its profound scientific significance. An important aspect of precision medicine is to deliver the right drug to the right patient at the right time and in the right dosage according to the unique characteristics of each patient, so as to maximize the efficacy of drugs and minimize adverse effects (Lou et al., 2023). At present, the difficulty in the treatment of cartilage injury clinical practice is the uncertain treatment efficacy and recovery cycle. MSCs-based alternative therapies are considered an effective treatment option for cartilage injury, and more and more researchers are turning their attention to the exploration and application of functional MSC subpopulations. This review mainly focus on analyzing the effects of three most widely used MSCs such as BM-MSCs, AD-MSCs and UC-MSCs in cartilage injury repair. Specifically, the effects of these MSCs are as follows: MSCs can differentiate into cartilage tissue and promote cartilage repair under effects of chondrogenic factors. In addition, MSCs can produce various ECMs during differentiation, including collagen, fibronectin, proteoglycans, GAGs, and a variety of cytokines, inhibit the degradation of ECMs, which is essential for the recovery of cartilage function. In the target repair region, MSCs can release a variety of cytokines, growth factors, and chemokines to drive endogenous MSCs into the injured region, attenuate the macrophage-induced intraarticular inflammation, and create a suitable regenerative microenvironment, thus promoting the regeneration of cartilage tissue (Figure 2). Other related ones such as synovial-derived MSCs (Sun et al., 2023; Mak et al., 2016) and chondrogenic progenitor cells (Wang H. C. et al., 2021) have been increasingly investigated.

MSCs differ in chondrogenic differentiation, immunomodulation, and matrix production due to their differences in donor, tissue origin, cell type, and even individual cells from which they are derived. Knowing as much as possible about the potentials of various MSCs for cartilage injury repair will help in the selection of appropriate progenitor cells, which will contribute to improving the accuracy of MSCs-based cartilage injury treatment, designing more effective and safer methods to treat cartilage injury, optimizing the utilization of MSCs, thus fully realizing their potential in the field of cartilage repair. However, MSCs-based therapies have inherent limitations, such as high risk of tumourigenesis, low retention rate, and stringent regulatory requirements in ethical aspect.

Recently, the paracrine factors of MSCs are becoming increasingly popular, especially EVs have been shown to play a role in restoring the biological functions of injured articular cartilage. MSC-EVs can enhance chondrocyte proliferation and matrix synthesis, weaken cell apoptosis, and regulate immune responses, leading to good new tissue filling and perfect integration with surrounding cartilage. The tissue morphology and matrix composition of the regenerated tissue are very similar to the native articular cartilage, making MSC-EVs a promising tool for soft tissue repair and treatment, achieving a therapeutic effect similar to that of MSCs in cartilage tissue engineering (Figure 2).

Although MSC-EVs are effective therapeutic agents for the treatment of articular cartilage injury and OA, the molecular mechanisms for their therapeutic effects, delivery routes, storage conditions and safety issues also demand further research and the establishment of corresponding standards (Nguyen et al., 2021). Fortunately, a team recently conducted the first human safety trial of UC-MSC-EV, showing no adverse events during the 12-month follow-up period after articular administration (Figueroa-Valdés et al., 2025). The clinical translation path of this study includes complete preclinical efficacy/safety assessment, production process standardization, and early clinical validation, providing a paradigm reference for advancing EV-based regenerative medicine product development.

Because the studies on the therapeutic effects of MSCs and their EVs are still in their infancy, and there is insufficient evidence to support the standard acquisition method of EVs, it is needed to perform further studies on EV isolation methods. So far, there are very few studies on the application of MSC-EVs in human beings, most studies focusing on the application of MSC-EVs in small animal models. The study subjects are mostly rats, mice, rabbits; a small number of studies on MSCs and MSC-EVs have been conducted in piglets and even advanced biological models. It is hoped that more large animal study models will be available in the future and eventually enter into clinical studies. With further research and clinical trials, future studies will help address challenges and ensure the safety and effectiveness of MSC-EVs, paving the way for their widespread application in the field of cartilage repair and potentially making them a powerful tool for cartilage injury treatment, thereby improving the quality of life for patients (Liao et al., 2024). Although EVs are not cells, their origination from cells may pose a challenge in terms of defining their legal classification and receiving approval from the FDA for use in the clinical setting (Krut et al., 2021). Once these considerations are addressed, clinical trials on the use of MSC-EVs for the treatment of Cartilage injury will be able to commence.

## Glossary

AD-MSCs: adipose tissue-derived MSCs
AFM: atomic force microscope
ApoEVs: apoptotic cell-derived EVs
BM-MSCs: bone marrow-MSCs
BMP: bone morphogenic protein
Col II: Type II Collagen
D cells: dendritic cells
DMM: destabilization of the medial meniscus
ECM: extracellular matrix
ERK: extracellular signal-regulated kinase
EVs: extracellular vesicles
FDA: food and drug administration
FGF-2: fibroblast growth factor 2
FTCDs: full-thickness cartilage defects
GAGs: glycosaminoglycans
HTO: high tibial osteotomy
hWJ-MSCs: human umbilical cord Wharton’s jelly-derived MSCs
ICRS: International Cartilage Repair Society
IFP-MSC: infrapatellar fat pad-derived MSC
ISCT: International Society for Cellular Therapy
ISEV: International Society for Extracellular Vesicles
ITGB1: integrin beta-1
KOA: knee osteoarthritis
LIPUS: low-intensity pulsed ultrasound
LT: light therapy
MAPK: mitogen-activated protein kinase
MIA: monoiodoacetate
MMP: matrix metalloproteinase
MRI: magnetic resonance imaging
MSC: mesenchymal stem cell
mTOR: mammalian target of rapamycin
NF-kB: NF-κB signal transduction pathway
NK cells: natural killer cells
NTA: nanoparticle tracking analysis
OA: osteoarthritis
OARSI: Osteoarthritis Research Society International
OAS: Oswestry Arthroscopy Score
OCDs: osteochondritis dissecans
PEMF: pulsed electromagnetic field
PI3K/Akt: phosphatidylinositol 3-kinase and protein kinase B
PRP: platelet-rich plasma
PTCDs: partial-thickness cartilage defects
PTOA: post-traumatic osteoarthritis
RFE: radiofrequency energy
ROS: reactive oxygen species
Runx2: recombinant runt related transcription factor 2
SMAD: *drosophila* mothers against decapentaplegic protein
SZ: superficial zone
TGF-β: transforming growth factor-β
Th1: helper T cell 1
TNF-α: tumor necrosis factor-α
UC-MSCs: Umbilical cord-derived MSCs
VEGF: vascular endothelial growth factor
WOMAC: Western Ontario and McMaster Universities Osteoarthritis index
